# Performance and carcass characteristics of Australian purebred and crossbred lambs supplemented with Rice Bran

**DOI:** 10.1186/s40781-015-0069-x

**Published:** 2015-10-01

**Authors:** Aaron Ross Flakemore, John Roger Otto, Bénédicte Suybeng, Razaq Oladimeji Balogun, Bunmi Sherifat Malau-Aduli, Peter David Nichols, Aduli Enoch Othniel Malau-Aduli

**Affiliations:** Animal Science and Genetics, Tasmanian Institute of Agriculture, School of Land and Food, Faculty of Science, Engineering and Technology, University of Tasmania, Private Bag 54 Sandy Bay, Hobart, TAS 7001 Australia; Institut National Supérieur des Sciences Agronomiques de l’alimentation et de l’environnmente (AGROSUP), 26 Bd du Docteur Petitjean, BP 87 999, 21079 Dijon Cedex, France; CopRice Feeds, PO Box 104, Cobden, VIC 3266 Australia; College of Medicine and Dentistry, Division of Tropical Health and Medicine, James Cook University, Townsville, QLD 4811 Australia; CSIRO Food and Nutrition, Oceans and Atmosphere Flagships, Hobart, TAS 7001 Australia; Veterinary and Biomedical Sciences, College of Public Health, Medical and Veterinary Sciences, Division of Tropical Health and Medicine, James Cook University, Townsville, QLD 4811 Australia

## Abstract

**Background:**

This study examined the effects of dietary supplementation with rice bran, sire breed and gender on live animal performance and carcass characteristics in Australian crossbred and purebred Merino lambs.

**Methods:**

Forty-eight lambs balanced by sire breed (Dorset, White Suffolk, Merino) and gender (ewe, wether) were randomly allocated into three dietary supplementation groups (Control- 24 lambs fed wheat/barley-based pellets, Low- 12 animals fed a 50/50 ratio of wheat-based/rice bran pellets, and High- 12 lambs fed rice bran pellets). The Rice bran pellets replaced 19 % of the barley component of the feed. Animals were group-fed at the rate of 1000 g of the supplement per head per day with *ad libitum* access to lucerne hay as the basal diet and water. The duration of the feeding trial was 49 days with an initial 21-day adjustment period.

**Results:**

Sire breed differences were evident for initial (*p* < 0.0002) and final (*p* < 0.0016) liveweights, hot carcass (*p* < 0.0030) and cold carcass (*p* < 0.0031) weights, as well as dressing percentage (*p* < 0.0078), fat thickness (*p* < 0.0467), yield grade (*p* < 0.0470) and rib eye area (*p* < 0.0022) with purebred Merino under-performing compared to the crossbreds. Concentrate feed conversion efficiency, costs per unit of liveweight gain and over the hooks income were comparable between treatments regardless of the observed trend where the high supplementation group tended to show lower feed intake (745.8 g/day) compared to both the control (939.9 g/day) and low supplementation groups (909.6 g/day). No significant differences (*p* > 0.05) were observed between treatments for live animal performance, carcass characteristics, gender and their second-order interactions.

**Conclusions:**

Results indicate that Rice bran can be utilised as a cost-effective supplementary feed source in genetically divergent sheep over a 49-day feeding period without detrimental effects on overall live animal performance or carcass characteristics.

## Background

Traditional concentrate supplementary feeds used by Australian sheep producers generally comprise wheat, barley and oats. However, these feed resources are subject to seasonal fluctuations and perturbations like drought and flooding which can impact on availability and uniformity of product quality. Likewise, increasing competition between humans and animals, animal industries, as well as increased production costs for these feeds escalate overall production costs for the sheep producer, thus impacting total on-farm profitability. Therefore, there is the need for readily available and cost-effective feed resources that are able to match or out-perform traditional supplementary feeds. These supplements should not only be cost effective, highly digestible and profitable, but also not impact negatively on animal health and wellbeing, the environment and social acceptability to the greater public [1, 2]. In this context, agro-industrial by-products such as rice bran (RB) have been put forward as viable options for ruminant production systems [3–5].

Rice bran, also referred to as rice pollard in Australia, is a by-product of rice milling that consists of the bran and polishings including the embryos, inner and outer bran layers, the starchy endosperm and traces of broken endosperm with relatively few hulls [4, 6, 7]. Nutritive analyses show that when stabilised, rice bran is an effective energy and fatty acid feed source constituting essential proportions of protein [6] and high content of oleic, linoleic and palmitic acids [4, 7, 8]. Rice bran is also rich in vitamins and minerals and is an exceptional source of gamma oryzanol which has both antioxidant and steroid-like properties [9].

Due to its relatively high unsaturated fatty acid content, the possibility of rice bran rancidity during storage exists [6, 10]. This can reduce animal feed intake with potentially negative effects on animal health and wellbeing. This has been the major limiting factor regarding the use of rice bran in animal production systems. Nevertheless, research has demonstrated that stabilised rice bran is an effective dietary energy and unsaturated fatty acid source for animals such as chickens, rats and pigs with relatively minor effects on growth and performance [10, 11]. Similarly, rice bran and its main components have demonstrated the ability to improve the plasma lipid pattern of rodents, rabbits, non-human primates and humans, reducing total plasma cholesterol and triacylglycerol concentrations and increasing high density lipoproteins [8].

A number of research articles[1, 3, 5, 12–15] have emerged evaluating rice by-products as dietary sources of nutrients in sheep production systems. However, to our current knowledge, since the late 1980’s and early 1990's research of [6, 7], there has been no other peer-reviewed published information on sheep supplementation with rice bran in Australian prime lamb production systems. This suggests that rice by-products have become relatively neglected feed constituents in Australian sheep production systems. Moreover, this lack of updated data represents a major knowledge gap given Australia’s predominance as one the world’s largest sheep producing nations [16], with an average production of 647kt of paddy rice from 2000 to 2013 for domestic and export consumption [17]. Therefore, the primary objective of this study was to assess the effect of dietary rice bran supplementation on live animal performance and carcass characteristics in genetically divergent purebred and crossbred lambs currently utilised by the Australian sheep industry. The hypothesis tested was that *rice bran can be used as an effective alternative supplementary feed resource for Australian sheep producers with prime lamb growth and carcass quality outcomes that are comparable to the traditional wheat-barley-based concentrate feeds*.

## Methods

### Animal ethics

The use of animals and procedures performed in this study were all approved by the University of Tasmania Animal Ethics Committee (Permit No A0013839) and were conducted in accordance with the 1993 Tasmanian Animal Welfare Act and the 2004 Australian Code of Practice for the Care and Use of Animals for Scientific Purposes.

### Experimental site and animal management

The feeding trial was conducted from 6^th^ May to 15^th^ July 2014 at the University of Tasmania Farm, Cambridge, Hobart, Tasmania, Australia. The animals were slaughtered at the Gretna Quality Meats Abattoir, Black Hills Road, Gretna, Tasmania, Australia on 17^th^ July 2014. Carcass dissection and meat quality parameter measurements were evaluated at Robinson Meats, Glenorchy, Hobart, Tasmania, Australia on 20^th^ July 2014.

The study was conducted using forty-eight (48), 8 month-old, weaned, purebred (*n* = 16) and crossbred (*n* = 32) prime lambs at an initial average body weight (BW) of 35.3 ± 4.3 kg. Lambs were progeny from Merino dams mated to Dorset, White Suffolk and Merino sires under the same management conditions. The experimental diets consisted of two iso-caloric and iso-nitrogenous wheat-based pellets. Table 1 shows the composition of experimental diets. Lucerne hay was used as the basal diet.

**Table 1:** Feed composition of the experimental diets

Ingredients %	Concentrate diet 1 (No rice bran)	Concentrate 2 (Added rice bran)
Wheat 12 %	25.00	25.00
Barley	25.87	-
Rice bran	-	18.97
GOMF	-	10.21
Mill mix	20.17	10.00
Lupins	16.00	14.81
Paddy rice	7.26	15.43
Limestone 37 %	2.09	1.96
Ammonium sulphate	1.25	1.25
Salt	1.00	1.00
Sodium bicarbonate	0.625	0.625
Acid buffer	0.625	0.625
Bovatec 20 %	0.01	0.01

Daily rates of 1000 g of the supplementary feeds per lamb were offered during the three-week adjustment period as a single meal between 0700–0900 h. After the adjustment period, the experimental supplements were provided as two equal meals, as per daily allocation on weight basis per animal in each dietary treatment at 0700–0900 h and 1500–1700 h daily. All animals had *ad libitum* access to lucerne hay and clean fresh water throughout the duration of the feeding trial. Concentrates were provided to animals via feeding troughs attached to one side of the fence in each feeding pen, with each trough providing enough access for up to three sheep at once. Lucerne hay was provided using an elongated low level feeding trough at a size of 1.8 m (length) × 125 mm (height) × 375 mm (width), thus allowing all lambs to feed simultaneously. The residual concentrate feeds from each supplementary feeding group was removed and weighed the following morning before the allocation of fresh rations. Lambs were housed indoors and group-fed according to their respective dietary allocations on open slatted wooden floors. Within each group, an average area of approximately 1 m^2^ per animal was provided. All animals were drenched against worms using Triguard (1 g L-1 Abamectin and 22.1 g L-1 Oxfendazole) according to the manufacturer’s recommendations.

### Experimental design and treatments

The trial utilised a completely randomised block design employing 3 sire breeds (Merino, Dorset and White Suffolk), 3 rice bran supplementation levels (Control, Low and High) and 2 genders (ewes and wethers). The three dietary treatments consisted of: a wheat-based concentrate pellet as the control; a diet consisting of 19 % rice bran replacing the barley component of the control pellet as the rice bran high diet (RBH); and a ration comprising a 50/50 combination of Control and RBH pelleted feeds as the rice bran low (RBL) diet. Dietary treatments consisted of 24 control animals, and 12 animals each of RBL and RBH. The feeding trial lasted 70 days, comprising a 21-day adjustment period, followed by 49 days of full supplementation.

### Feed analysis

Representative samples of the experimental feeds and lucerne hay collected from each bale were used for chemical analysis. The experimental feeds were finely ground to pass through a 2 mm sieve using Laboratory Mill (Thomas Model 4 Wiley® Mill; Thomas Scientific). Dry matter (DM) and moisture content (MC) were determined by drying samples at 105 °C for 24 h. Ash content was determined by combusting samples in a furnace at 600 °C for 8 h. Neutral Detergent (NDF) and Acid Detergent (ADF) fibre contents were measured using an Ankom Fibre Analyzer (ANKOM220; ANKOM Technology, USA). Nitrogen content was determined using a Thermo Finnigan EA 1112 Series Flash Elemental Analyzer and the values multiplied by 6.25 to give the crude protein (CP) percentage. Ether extract (EE) was determined using an Ankom fat/oil extractor (ANKOMXT15; ANKOM Technology, USA). Total digestible nutrients (%TDN) were calculated as %TDN = 88.9 - (ADF% × 0.779). Metabolisable energy (ME) was calculated by converting %TDN to digestible energy (DE [Mcal/kg] = %TDN × 0.01 × 4.4) which was converted as ME = (DE (Mcal/kg) × 0.82) × 4.185. Table 2 shows the chemical compositions and energy values of the experimental and basal diets.

**Table 2:** Proximate analysis of the experimental and basal diets

Chemical composition (%DM)	Feed components			
	Lucerne	Control	Rice bran low	Rice bran high
MC	10.4	10.2	8.0	9.8
DM	89.6	89.8	92.0	90.2
ADF	27.4	8.2	8.6	8.8
NDF	46.5	23.8	21.1	20.0
EE	1.1	2.8	3.4	4.0
Ash	5.3	3.4	6.4	8.1
CP	17.3	15.0	14.0	12.0
%TDN	67.6	82.5	82.2	82.0
ME (MJ/kg)	10.2	12.5	12.4	12.4

Feeds were analysed on a dry matter basis; Moisture content (MC), Dry matter (DM), Neutral detergent fibre (NDF), Acid detergent fibre (ADF), Ether extract (EE) and crude protein (CP), Total digestible nutrients (%TDN), Metabolisable energy (ME)

### Liveweight and feed intake

Individual animal liveweights were recorded at weekly intervals prior to morning feeding. An electronic Tru-Test XR3000 livestock Walk-Over Weighing (WOW) system was used with animals standing in a relaxed position. Average daily gain was calculated as the difference between initial and final weights divided by the number of days of supplementation. Average concentrate feed intake per animal was calculated as the total feed allocated minus the residual feed divided by the number of animals for that treatment group. Feed conversion efficiency was calculated as the average daily feed intake (g)/1000 × 49 [days of supplementation]/Total weight gain (kg) over the full trial period. Concentrate cost per kg of live animal weight gain was calculated as concentrate feed conversion efficiency × ($/t × 1000 kg) of supplementary feed. Feed costs ($/kg) were based on an average price of pellet manufacture at $AU 406/t, $AU 379/t and $AU 352/t for control, RBL and RBH diets respectively.

### Slaughter protocol and carcass data

Forty lambs (minus the 8 purebred Merino ewes retained for breeding purposes) were transported to Gretna Quality Meats abattoir in the morning after the final day of the feeding trial. The animals remained in lairage for approximately 24 h and were slaughtered adhering to Australian guidelines and practices for the humane sacrifice of livestock in commercial operations. After 24 h chilling, carcasses were transported in a refrigerated truck for 45 min to Robinson Meats Glenorchy for commercial cutting and carcass measurements. Measurements of hot carcass weights (HCW) were taken after evisceration before carcass chilling. Cold carcass weights (CCW) were recorded 24 h thereafter. Dressing percentage (%) was calculated as = (HCW/initial liveweight) × 100. Carcass measurements of fat thickness and body wall thickness were taken at the 12th and 13th rib interface using a GR knife over the mid-point of the *Longissimus dorsi* muscle perpendicular to the outside surface of the fat. Body wall thickness was measured 11 cm from the centre of the spine using tissue depth criteria as outlined on the GR fat knife. Rib eye area (REA) of the *Longissimus dorsi* muscle was evaluated using a plastic grid. Yield grade was determined as 0.4 + (10 × fat depth). % Boneless, Closely Trimmed Retail Cuts (BCTRC) was calculated as 49.936 - (0.0848 × hot carcass weight) - (4.376 × fat depth) - (3.530 × body wall thickness) + (2.456 × REA). Over the hooks (OTH) trade value was calculated as HCW × 500¢/kg divided by 100¢ to provide an average total dollar value per carcass for animals from each treatment group. 500¢/kg was the amount received per kg for the sale of the lambs used in this study, and is within the range for OTH prices for 2014 [18]. All values were calculated in Australian dollars.

### Statistical analysis

Statistical analyses of data were performed using Statistical Analysis System [19]. Summary statistics by supplementation level, sire breed and gender were computed using PROC MEANS. The General Linear Model procedures (PROC GLM) were used for multi-trait analysis of variance fitting the fixed effects of supplementation level, sire breed and gender and their second-order interactions. Significant pairwise comparisons and mean separations set at a minimum threshold of *p* < 0.05 level were carried out using Duncan’s and Tukey’s tests for fixed effects and interactions, respectively. Due to the group feeding design of this trial statistical evaluation of variance, feed intake, concentrate feed conversion efficiency and feed cost per unit gain were presented as group averages as per Vipond et al. [20].

## Results

### Feed analysis

Proximate analysis of the experimental diets is presented in Table 2. The DM content was comparable between all dietary treatments at ~90 %DM. ADF content for the lucerne at 27.4 %DM was up to three fold higher than that of the concentrates which were 8.2, 8.6 and 8.8 %DM for the Control, RBL and RBH diets, respectively. NDF content was also greater for lucerne at 46.5 %DM compared to 23.8, 21.1 and 20.0 %DM for the control, RBL and RBH, respectively. Ether extract and Ash contents were both greater for the RBH concentrate (4.0 %DM and 8.1 %DM) compared to the Control (2.8 %DM and 3.4 %DM), RBL (3.4 %DM and 6.4 %DM) and lucerne (1.1 %DM and 5.3 %DM). The Crude Protein content of the RBH concentrate at 12.0 %DM was lower than all other feeds at 17.3%DM, 15.0 %DM and 14.0 %DM for Lucerne, Control and RBL respectively. Total digestible nutrients, digestible energy (Mcal/kg) and metabolisable energy (MJ/kg) were comparable between the supplementary concentrate diets at 82 %TDN, 3.6 Mcal/kg and 12.5 MJ/kg, respectively. The basal lucerne diet was comparatively lower in %TDN, DE and ME (67.6 %, 3.0 Mcal/kg and 10.2 MJ/kg, respectively).

### Rice bran supplementation

Initial and final body weights (BW) of 35.8 ± 0.91 and 44.5 ± 1.09, 33.9 ± 1.19 and 44.5 ± 1.8, and 35.6 ± 1.2 and 43.4 ± 1.8 for the Control, RBL and RBH diets, respectively, were comparable between treatments (*p* > 0.05) as depicted in Table 3. Treatment differences in total and average daily gains were also not statistically significant (*p* > 0.05). However, in absolute terms, RBL tended to be higher (10.58 ± 1.19 kg) in total weight gain over the trial period compared to the control (8.7 ± 0.5 kg) and RBH (7.8 ± 1.0 kg) diets. These equated to average daily gains of 216.0 ± 24.2 g/day, 177.3 ± 10.2 g/day, and 159.0 ± 19.6 g/day for RBL, Control and RBH diets, respectively.

**Table 3:** Means and standard errors (M ± SE) of liveweight, average daily gain and dry matter intake of prime lambs supplemented with rice bran

	Control	Rice bran low	Rice bran high	*P* value
Initial BW (kg)	35.8 ± 0.91	33.9 ± 1.19	35.6 ± 1.2	0.3441^ns^
Final BW (kg)	44.5 ± 1.09	44.5 ± 1.84	43.4 ± 1.8	0.8359^ns^
Total weight gain (kg)	8.7 ± 0.50	10.6 ± 1.19	7.8 ± 1.0	0.1253^ns^
Av. Daily gain (g/day)	177.3 ± 10.24	216.0 ± 24.2	159.0 ± 19.6	0.1253^ns^
Supp. Feed intake (g/day)^a^	939.9	909.6	745.8	0.0553^ns^
FCE^b^	5.3	4.2	4.7	0.1523^ns^
FCPUG^c^	2.2	1.7	1.9	0.0653^ns^

^a^Supp. Feed intake (g/day) is based on the average intake per group divided by the number of animals per feeding group over the period of full supplementation (days 21–70). ^b^FCE = Concentrate feed conversion efficiency (kg concentrate/kg BW per animal). ^c^FCPUG = Feed cost per unit gain (Concentrate cost of feed/kg live weight gain ($AU/kg) per animal). Level of significance: ns not significant (*p* > 0.05)

Lambs subjected to the RBH supplementation diet consumed less concentrate compared to the Control and RBL diets, at an average intake of 745.8 g/day, compared to 939.9 g/day and 909.6 g/day, respectively, although these values narrowly missed statistical significance at *p* > 0.0553 (Table 3). Feed conversion efficiency (FCE) was comparable between supplementation levels at 5.3 (kg/kg BW), 4.2 (kg/kg BW), and 4.7 (kg/kg BW) for Control, RBL and RBH diets respectively (Table 3). This consecutively reflected no differences in concentrate costs per kg of live weight gain per animal between concentrate treatments of $AU 2.2/kg, $AU 1.7/kg and $AU 1.9/kg for Control, RBL and RBH respectively.

The inclusion of RB in the concentrate had no significant (*p* > 0.05) influence on any of the carcass characteristics measured in this study (Table 4). This resulted in over the hooks (OTH) trade showing no significant differences in income returned per carcass between treatments. However, average income from RBH supplemented lambs at $AU 97.00 ± 7.15 was lower per animal than both RBL ($AU 102.15 ± 5.57) and the Control ($AU 106.25 ± 3.75). This equated to RBH differences of -$AU 5.15 and -$AU 9.25 compared to RBL and Control lambs respectively, and a difference of $AU −4.10 between RBL and the Control.

**Table 4:** Effect of treatment on carcass characteristics (Least squares means ± SEM)

	Control	Rice bran low	Rice bran high	*P* value
Pre-slaughter weight (kg)^a^	46.0 ± 1.1	46.0. ± 1.8	44.2 ± 2.1	0.3150^ns^
HCW (kg)^b^	21.3 ± 0.8	20.4 ± 1.1	19.4 ± 1.4	0.3609^ns^
CCW (kg)^c^	21.0 ± 0.7	20.1 ± 1.1	19.1 ± 1.4	0.3417^ns^
Dressing percentage (%)	46.2 ± 1.2	44.4 ± 1.6	43.5 ± 1.5	0.6819^ns^
Fat thickness (mm)	4.5 ± 0.3	4.3 ± 0.5	3.8 ± 0.5	0.1602^ns^
Body wall thickness (mm)	15.2 ± 0.7	17.1 ± 1.1	14.2 ± 1.3	0.1639^ns^
GR fat score (1–5)	3.5 ± 0.2	4.0 ± 0.3	3.1 ± 0.3	0.0912^ns^
Yield grade	2.2 ± 0.1	2.1 ± 0.2	1.9 ± 0.2	0.1596^ns^
Rib eye area (cm^2^)	15.0 ± 0.5	15.3 ± 0.8	14.5 ± 0.9	0.5246^ns^
BCTRC%^d^	48.8 ± 0.2	48.8 ± 0.3	49.2 ± 0.4	0.4228^ns^
OTH trade ($AU)^e^	106.3 ± 3.8	102.2 ± 5.6	97.0 ± 7.2	0.4398^ns^

^a^Pre-slaughter weight is the weight of animals (minus Merino ewes) prior to transport for slaughter. ^b^HCW = Hot carcass weight. ^c^CCW = Cold carcass weight. ^d^BCTRC% = Boneless, Closely Trimmed Retail Cuts. ^e^OTH = Over the hooks trade (this was based on 500¢AU return per kg of HCW). Level of significance: ns not significant (*p* > 0.05)

### Sire breed

Terminal sire breed effects (Table 5) for both initial (*p* < 0.0002) and final live weights (*p* < 0.0016) showed that purebred Merino lambs were significantly lighter than Dorset and White Suffolk crosses. Merino lambs weighed 31.8 ± 0.9 kg and 40.1 ± 1.1 kg, compared to Dorset at 37.3 ± 1.1 kg and 46.8 ± 1.6 kg, and White Suffolk at 36.7 ± 0.6 kg and 45.7 ± 1.0 kg for initial and final weights, respectively. Total weight gain and average daily gains were not dependent upon the influence of terminal sire breed (*p* > 0.05). No significant (*p* > 0.05) sire breed interactions with RP supplementation level or gender were identified. Therefore, these interaction results are not presented.

**Table 5:** Means and standard errors (M ± SE) of live animal performance and carcass characteristics of prime lamb progeny from different sire breeds

	Dorset	White Suffolk	Merino	*P* value
Initial BW (kg)	37.3 ± 1.1	36.7 ± 0.6	31.8 ± 0.9	0.0002***
Final BW (kg)	46.8 ± 1.6	45.7 ± 1.0	40.1 ± 1.1	0.0016**
Total weight gain (kg)	9.5 ± 1.0	9.0 ± 0.7	8.3 ± 0.7	0.4418^ns^
Av. Daily gain (g)	194.5 ± 20.3	183.7 ± 14.5	169.0 ± 14.8	0.4418^ns^
Pre-slaughter weight (kg)^c^	47.0 ± 1.6	45.7 ± 1.0	42.2 ± 1.78	0.0540^ns^
HCW (kg)^d^	22.0 ± 0.9^a^	21.3 ± 0.8^a^	16.5 ± 0.9^b^	0.0030**
CCW (kg)^d^	21.7 ± 0.9^a^	21.0 ± 0.8^a^	16.2 ± 0.9^b^	0.0031**
Dressing percentage (%)	46.7 ± 0.8^a^	46.3 ± 1.2^a^	39.2 ± 1.7^b^	0.0078*
Fat thickness (mm)	4.7 ± 0.4^a^	4.3 ± 0.3^a^	3.3 ± 0.5^b^	0.0467*
Body wall thickness (mm)	16.1 ± 1.1	15.6 ± 0.7	13.8 ± 1.2	0.2648^ns^
GR fat score (1–5)	3.7 ± 0.3	3.6 ± 0.2	3.1 ± 0.3	0.3013^ns^
Yield grade	2.3 ± 0.5^a^	2.1 ± 0.1^a^	1.7 ± 0.8^b^	0.0470*
Rib eye area (cm^2^)	15.6 ± 0.5^a^	15.5 ± 0.5^a^	12.3 ± 0.8^b^	0.0022**
BCTRC %^e^	48.7 ± 0.2	49.0 ± 0.2	49.0 ± 0.3	0.5937^ns^
OTH^f^	109.8 ± 4.2^a^	106.3 ± 3.9^a^	82.5 ± 4.6^b^	0.0030**

^c^Pre-slaughter weight is the weight of animals (minus Merino ewes) prior to transport for slaughter. ^d^HCW = Hot carcass weight. ^e^CCW = Cold carcass weight. ^f^BCTRC% = Boneless, Closely Trimmed Retail Cuts. ^f^OTH = Over the hooks trade (this was based on 500¢ AU return per kg of HCW). Level of significance: ns not significant (*p* > 0.05), * significant (*p* < 0.05), ** highly significant (*p* < 0.01), and *** very highly significant (*p* < 0.001). Different superscripts indicate significant differences within each row (*p* < 0.05)

Both HCW (*p* < 0.0030) and CCW (*p* < 0.0031) were significantly influenced by sire breed, with the purebred Merino showing lower weights compared to both Dorset and White Suffolk crossbred lambs. This is reflected by significant differences (*p* < 0.003) in OTH income with both Dorset ($AU109.8 ± 4.2) and White Suffolk ($AU106.3 ± 3.9) crossbreds higher in average income per carcass than purebred Merino ($AU82.5 ± 4.6). Differences in dressing % (*p* < 0.0078), Yield grade (*p* < 0.0470) and REA (*p* < 0.0022) were also significant with Dorset and White Suffolk sired progeny having higher values in these categories compared to the Merino. The Merino also displayed significantly lower fat thickness (*p* < 0.0467) compared to other sire breeds. There was no significant difference in body wall thickness (*p* > 0.05) or GR fat score (*p* > 0.05).

### Sex

There were no significant (*p* > 0.05) differences between ewe and wether lambs for liveweight, carcass traits or OTH income (Table 6). However, in absolute terms, wethers tended to produce live responses that were marginally superior to those of ewes, with slightly leaner carcasses. Interaction effects between gender and supplementation level were all non-significant, hence not presented in tables.

**Table 6:** Means and standard errors (M ± SE) of live animal performance and carcass characteristics of ewe and wether prime lambs

	Ewe	Wether	*P* value
Initial BW (kg)	34.7 ± 0.89	35.9 ± 0.86	0.4320 ns
Final BW (kg)	42.9 ± 1.1	45.5 ± 1.18	0.1336 ns
Total weight gain (kg)	8.2 ± 0.5	9.8 ± 0.75	0.1202 ns
Av. Daily gain (g)	167.5 ± 11.0	197.3 ± 15.29	0.1202 ns
Pre-slaughter weight (kg)^a^	45.5 ± 1.2	45.5 ± 1.18	0.3318 ns
HCW (kg)^b^	21.0 ± 0.7	20.3 ± 0.87	0.3662 ns
CCW (kg)^c^	20.7 ± 0.7	20.0 ± 0.87	0.3738 ns
Dressing percentage (%)	46.1 ± 0.9	44.4 ± 1.19	0.8343 ns
Fat thickness (mm)	4.7 ± 0.3	4.0 ± 0.27	0.3389 ns
Body wall thickness (mm)	15.9 ± 1.1	15.1 ± 0.62	0.6601 ns
GR fat score (1–5)	3.6 ± 0.3	3.5 ± 0.16	0.6415 ns
Yield grade	2.2 ± 0.1	2.0 ± 0.10	0.3385 ns
Rib eye area (cm2)	15.2 ± 0.5	14.8 ± 0.51	0.4659 ns
BCTRC %^d^	48.8 ± 0.2	49.0 ± 0.17	0.6550 ns
OTH^e^	104.9 ± 3.4	101.6 ± 4.3	0.3662 ns

^a^pre-slaughter weight is the weight of animals (minus Merino ewes) prior to transport for slaughter. ^b^HCW = Hot carcass weight. ^c^CCW = Cold carcass weight. ^d^BCTRC% = Boneless, Closely Trimmed Retail Cuts. ^e^OTH = Over the hooks trade (this was based on 500¢ AU return per kg of HCW). Level of significance: ns not significant (*p* > 0.05)

## Discussion

### Proximate analysis of feeds

The 89.6 %DM, 17.3 %CP and 10.2 ME contents of the lucerne hay used as the basal diet in this study were similar to the 87%DM, 18%CP and 9 (MJ/kg) averages for Australian lucerne hay [21]. The CP content of the basal diet was in excess of the 7 % CP content required in feeds to support acceptable rumen microbial activity and the maintenance requirement of the host ruminant [22]. The high ADF content of the lucerne reduced the %TDN and energy values compared to the concentrates. The ME value of 10.2 MJ/kg in the basal lucerne hay was lower than the 12 MJ/kg required in a ration for ideal growth rates [23]. This indicates that supplementary feeding was required, and that any observed effects on growth performance were more likely a response to the addition of the concentrate supplementary feeds.

The DM, CP and ME values for RBL and RBH were comparable to the averages for Australian RB reported by Hinton [21] and Warren and Farrell [7]. The CP contents of both RB containing diets used in the present study were in excess of the 7 % CP requirement for ruminant maintenance. CP was higher than those of Nega and Melaku [14] and Asmare et al. [3] at 11 % and 7.8 % respectively, but comparable to the 15 % and 13 % in the RB diets of Tabeidian and Sadeghi [5]. The RB diets in the present study contained less than the 25.6 % NDF, 12.2 % ADF, 10.8 % Ash and 22 % EE values reported by Warren and Farrell [7]. The discrepancies are reflective of the report of Warren and Farrell [7] that despite the quality of Australian produced rice bran being reasonably uniform, differences in nutritive value are mainly due to variations in seasonal conditions and cultivar variety.

### Rice bran supplementation

The non-significant differences in animal performance and carcass characteristics associated with rice bran supplementation supports our tested hypothesis. This confirms that rice bran can be used as an effective supplement at levels of up to 19 % without impeding the ability of lambs to achieve a targeted 45 kg liveweight, ideal for the Australian domestic market from an initial 35 kg liveweight when fed over a 49-day full dietary finishing period.

The results in this study compare to those previously reported by Tabeidian and Sadeghi [5] showing no significant differences in overall live animal performance with rice bran replacing barley at levels of up to 30 % in a concentrate diet fed to uncastrated male Afshari lambs over a period of 85 days. However, whilst the initial liveweights herein were comparable to those of Tabeidian and Sadehi [5], the final, average daily and total weight gains were extensively lower. This may be due to a combination of factors between studies; breed, gender, age, feeding duration and management conditions. The average daily gains are similar to those reported by Hogan et al. [6] in which Border Leicester × Merino × Dorset ewe and wether lambs administered rice bran oil at 10 % inclusion level achieved growth rates between 100–150 g/day under Australian production systems. Similarly, the improved liveweight gains for the RBL treatment compared to the Control and RBH diet in this study agree with the findings of Asmare et al. [3], Nega and Melaku [14] and Salinas-Chavira et al. [24] demonstrating that mixed ration formulations with rice by-products improved and/or maintained live animal performance attributes in sheep.

Feed intake declined with rice bran at an inclusion level of 19 % in this study. This was unexpected given previous findings that total feed intake (hay + concentrate) (at ~1500 g/day) was generally unaffected when lambs were fed rations containing up to 30 % rice bran in the finishing diet [5], or in dairy goats fed 20 % rice bran [25]. Likewise, Hogan et al. [6] demonstrated that Australian extracted rice bran oil at levels of 10 % inclusion did not generally affect feed intake of Australian crossbred sheep averaging 1 kg/day. However, the decline in feed intake in this study agrees with the work of Tabeidian and Sadeghi [5] demonstrating that total feed intake (concentrate + hay) declined in sheep fed concentrates containing rice bran at levels of 45 % (1330 g/day) and 60 % (1150 g/day) compared to 0 % (1450 g/day). Likewise, Bhatt et al. [12] showed that in Malpura lambs offered ~1500 g/day, the concentrate intake significantly declined from 550 g/day to 472 g/day when 40 g/kg of rice bran oil was added.

Numerous mechanisms for the occurrence of reduced feed intake associated with RB had been proposed. Asmare et al. [3] and Cutrim et al. [13] advocated that reduced intakes with rice bran supplemented sheep are due to high NDF and ADF contents of RB. Whereas Park et al. [25] suggested that reduced feed intake was associated with lower production of acetate and ß-hydroxy-butyrate in the rumen or due to increased uptake of dietary long-chain fatty acids, thus inhibiting *de novo* fatty acid synthesis. Garg et al. [26] outlined that concentrates containing high levels of rice bran affected the digestibility of fat, protein and ADF in the rumen. Both Boucque and Fiems [4] and Nega and Melaku [14] suggested that elevated mineral content, namely increased levels of silica, was the main limiting factor affecting dietary intake of rice bran in sheep when fed in large amounts. The diets used in this study showed comparable levels of CP, EE, NDF and ADF between treatments. Therefore, it is likely that the major factor that affected dietary intake was the relatively high NDF and mineral contents of the RBH diet compared to the other treatments [13].

The similar carcass characteristics between treatments in this study demonstrates that rice bran can be used as an effective alternative supplementary feed source for Australian sheep producers. This overall finding again supports the tested hypothesis. However, the minor differences in body wall thickness between treatments corresponded to differences in GR fat score. Nevertheless, given that all dietary treatments produced sheep with carcass characteristics that meet the majority of Australian domestic and export market specifications for sheep meat [27], it is unlikely that any differences in GR fat class would translate into substantial differences in potential sale value.

Previously published investigations assessing the impact of rice bran on carcass characteristics have demonstrated comparable results to those presented herein. Tabeidian and Sadeghi [5] showed that RB at levels of up to 65 % in the concentrate diet of sheep had no effect on slaughter weight or HCW. However, they reported that a significant increase in dressing percentage occurred when sheep were supplemented with RB at 15 % compared to the control. These authors could not attribute this observation to any particular causal effect. However, given that only three lambs from each dietary group were slaughtered in their trial, it is our opinion that this could have been a response to individual variation between experimental groups. Nevertheless, no differences in dressing percentage between RB levels or the control were observed when sheep were supplemented with RB at levels of 30, 45 and 60 %. This was despite significant poorer animal performance particularly at the 45 % and 60 % inclusion level. Similar to our results, Salinas-Chavira et al. [24] showed no significant differences in *Longissimus dorsi* muscle area between treatments with 18 % rice polishings and tallow compared to the basal diet fed as a supplement to Pelibuey lambs. In another study, Bhatt et al. [12] showed similar dressing yields, loin eye areas and body fat distributions between 4 % RBO supplemented sheep compared to a concentrate control diet, but their study was marked by significantly lower HCW between treatments.

Studies observing reduced feed intakes with minimal effects on live animal performance have led to recommendations that the application of rice by-products in sheep diets may be more advantageous from an economic viewpoint [1, 13]. The reduced feed intakes between treatments, with no differences in feed requirements, or costs per kg of live weight gain between treatments herein concur with this line of thinking. However, the possible variable income between animals of differing treatments, whilst not significant would show considerable differences in overall flock income. The extent of cost-benefit analysis in feed conversion ratio and feed per kg live weight gain are dependent upon price differentiations between feed sources, rice bran quality and composition, application rate, supplement intake, and OTH prices offered at the time of sale. Other management input considerations such as time, labour, infrastructure costs and total on-farm profitability margins should also be considered.

### Sire breed

Results indicate that the greatest variations in animal performance for this study are derived between sire breeds. Similarly, the lack of significant interactions between sire breed and gender, and sire breed and supplementation level indicates that genetic performance through the influence of sire breed is maintained regardless of these influences. This outcome is in line with previous research findings that the Merino is a slower growing, leaner sheep breed, taking longer to reach maturity and therefore market slaughter weights [28–31]. This is particularly evident when compared to other breeds and 1^st^ cross lambs currently used in Australian sheep production systems.

The lack of significant differences in average daily gains between sire breeds in this study is comparable to Holman et al. [32], who also showed no significant differences between these breeds and crosses when trialling *Spirulina* supplementation. It is established that the Merino has a genetic predisposition for increased wool growth compared to both Dorset and White Suffolk, both with biases for growth and muscling [30, 33]. Therefore, it can be reasoned that similarities in gains between breeds was primarily a product of combined effects of wool production and body weight increases in the Merino. In comparison, increases in BW for lambs from Dorset and White Suffolk 1^st^-crosses were primarily a reflection of the genetic predispositions of these sire breeds for growth.

When purebred Merino lambs are slaughtered at the same age as other breeds, notably 1^st^ cross lambs sired from established meat breeds, the Merino exhibits significantly poorer carcass performance characteristics [28, 30, 33]. This consequentially manifests in lower rates of returns for straight Merino carcasses. This further indicates that nutrient partitioning between animals based upon genetic variation and predispositions for production traits are prevalent amongst the breeds utilised in this study.

### Sex

Similar performance between ewes and wethers regardless of nutritional or sire breed influences indicates that whilst male lambs are inherently larger and leaner, castration reduced male hormonal effects thereby resulting in wethers showing growth and carcass compositions comparable to those of ewes [34, 35]. This provides marketing options for producers regarding the use and management of ewe and wether lambs in their flock.

## Conclusions

This study is of importance as it is, as far as we are aware, the first in over two decades to evaluate the effects of rice bran supplementation for lambs used by the Australian sheep industry. Additionally, the results of the study are of importance as they have further added to current knowledge of the impact of RB as a supplementary feed source on the productive performance of purebred and crossbred sheep. In summary, supplementation of prime lambs with RB at an inclusion level of 19 % to replace barley in a concentrate diet did not negate live animal performance or carcass characteristics over a 49-day finishing period. Furthermore, RB was shown as an effective supplementary feed in terms of costs to the prime lamb producer. Purebred Merino lambs at the same age and management conditions as crossbreds demonstrated comparatively lower live performance and carcass characteristics. Production performance was similar between ewes and wethers. Put together, these results will enable sheep producers to make informed management decisions regarding the utilisation of RB in their flock for finishing prime lambs.
